# Integrating water depth to predict the threshold of collapse and recovery of submerged macrophytes for lakes with large depth gradients

**DOI:** 10.3389/fpls.2025.1541394

**Published:** 2025-02-24

**Authors:** Yexin Yu, Yehao Li, Haijun Wang, Haojie Su, Qingyang Rao, Ying Liu, Ping Xie

**Affiliations:** ^1^ Yunnan Key Laboratory of Ecological Protection and Resource Utilization of River-Lake Networks, Institute for Ecological Research and Pollution Control of Plateau Lakes, School of Ecology and Environmental Science, Yunnan University, Kunming, China; ^2^ Institute of International Rivers and Eco-Security, Yunnan University, Kunming, China; ^3^ Institute of Yunnan Plateau Indigenous Fish, Kunming, China; ^4^ Donghu Experimental Station of Lake Ecosystems, State Key Laboratory of Freshwater Ecology and Biotechnology, Institute of Hydrobiology, Chinese Academy of Sciences, Wuhan, China

**Keywords:** submerged macrophytes, thresholds, water depth, nutrient, turbidity, transparency

## Abstract

**Introduction:**

The nutrient threshold of collapse and recovery of submerged macrophytes have been widely reported for shallow lakes. However, understanding the threshold variation for lakes with water depth (Z) gradients remains limited.

**Methods:**

In this study, based on a field investigation of 9 lakes with varying water depths and nutrient levels in the Yunnan Plateau, southwest of China, we integrated water depth to predict the nutrient threshold of collapse and recovery of submerged macrophytes in lakes.

**Results:**

Our results showed that: 1) Canopy-forming submerged macrophytes, i.e. *Potamogeton pectinatus* and *Myriophyllum spicatum*, had a higher resistance to high nutrients and turbidity; 2) Submerged macrophyte species richness had a significantly negative response to water depth, while biomass did not; 3) A multiplication of turbidity (Turb) with water depth provided the best explanation for the collapse and recovery of submerged macrophytes for lakes with large depth gradients compared to the single variables; 4) The thresholds of Z_SD_/Z were 0.06 for the collapse of submerged macrophytes and 0.53 for the recovery of submerged macrophytes; the corresponding thresholds were 81.6 and 9.92 NTU m for Turb*Z, respectively.

**Discussion:**

Our findings on the thresholds of macrophyte collapse and recovery are expected to provide quantitative guidance for lake restoration of diverse water depths.

## 1 Introduction

The extinction of submerged macrophytes in lakes has become a global ecological problem (Dakos et al., 2019; Carpenter et al., 2022), and the explored triggering factors varied from nutrient loading (Ibelings et al., 2007; Shang et al., 2023), fish introduction (Zambrano et al., 2001; Hobbs et al., 2016), changes in hydrological regime (Søndergaard et al., 1992; Wu et al., 2013), to climate changes (Scheffer et al., 2001b; Moss et al., 2011; Zhang et al., 2021). The disappearance of submerged macrophytes is often unpredictable, catastrophic, and irreversible (Scheffer et al., 2001a; Dakos et al., 2015; Janssen et al., 2021), posing a great threat to water quality and the survival of other aquatic organisms for a long time (Carpenter and Lodge, 1986; Jeppesen et al., 1998; Scheffer and Carpenter, 2003).

The growth of submerged macrophytes is often affected by multiple factors (Middelboe and Markager, 1997; Azzella et al., 2014; Zhang et al., 2017; Ren et al., 2022). Light has been most frequently reported as the main limiting factor affecting submerged macrophyte growth (Madsen et al., 2001). Generally, submerged macrophytes can only survive at depths where light intensity reaches at least 1% of that on the water surface (Sculthorpe, 1967). In many eutrophic lakes, the lack of light at the lake bottom and competition for light in the water column by phytoplankton resulted in the disappearance of submerged macrophytes (Blindow et al., 2006). Water depth affected the distribution of submerged macrophytes (May and Carvalho, 2010). Many studies have shown that deep water can attenuate underwater light intensity and inhibit the growth and spread of submerged macrophytes (Søndergaard et al., 2013). The abundance and maximum distribution depth of submerged macrophytes in deeper lakes were strongly related to light conditions (Chambers and Kalff, 1985; Sand-Jensen and Madsen, 1991). Besides, submerged macrophytes with different morphologies and physiologies are distributed in different water depth ranges (Fu et al., 2014; Wen et al., 2022). Excessive nutrient input caused the overgrowth of phytoplankton. The latter reduced the underwater light through the shading effect, thus impeding the survival of submerged macrophytes (Madsen et al., 2001; Sayer et al., 2010; Yu et al., 2015; van Wijk et al., 2023).

The nutrient thresholds of collapse and recovery of submerged macrophytes were originally proposed and widely studied for shallow lakes. Studies included multi-lake comparison (Jeppesen et al., 1990; Kosten et al., 2009; Wang et al., 2014), long-term monitoring (Jeppesen et al., 1999; Ibelings et al., 2007), and paleolimnological studies (Yang et al., 2006). A long-term observation of Lake Veluwe in the Netherlands showed that the coverage of aquatic plants decreased gradually when the total phosphorus (TP) increased to 0.15 mg L^-1^, and disappeared completely at a TP concentration higher than 0.20 mg L^-1^ (Ibelings et al., 2007). Data on water parameters and biotic factors from the 1950s to 2009 in Lake Dianchi identified that the TN and TP concentration thresholds at which the collapse of submerged macrophytes occurred were 1.2 mg L^-1^ and 0.13 mg L^-1^, respectively (Wang et al., 2018). A multi-lake analysis including empirical data from subtropical lakes on the Yangtze floodplain found that the TP thresholds of collapse and recovery of submerged macrophytes were 0.08-0.12 mg L^-1^ and 0.04-0.06 mg L^-1^, respectively (Wang et al., 2014). Besides, other factors, such as transparency and water depth, also had limited impact on submerged macrophytes. An empirical model between submerged macrophytes and the ratio of Secchi depth (Z_SD_) to mean depth (Z_M_) in Yangtze lakes found that when the Z_SD_/Z_M_ threshold was less than 0.45, the disappearance of submerged macrophytes may occur (Wang et al., 2005). Although most of these thresholds were widely based on shallow lakes, understanding of nutrient thresholds for lakes with large depth gradients is still very limited.

Studies based on simulation models suggested that thresholds of nutrients and critical turbidity for the thresholds of collapse and recovery of submerged macrophytes generally decreased with water depth (Genkai-Kato and Carpenter, 2005; Scheffer and van Nes, 2007; Janse et al., 2008). An empirical study based on multi-lake comparison and long-term monitoring for the Yangtze shallow lakes revealed that TP thresholds vary little at moderate depths, with an assumed notable decrease when depth exceeds a level of probably 3-4 m (Wang et al., 2014). The distribution and growth of submerged macrophytes are closely linked to water depth, it normally acted together with underwater light conditions and nutrients to influence the growth of submerged macrophytes (Middelboe and Markager, 1997; Baastrup-Spohr et al., 2016; Chou et al., 2022; Zhang et al., 2022a). At specific eutrophication status, deeper distribution means stronger stress of light limitation on submerged macrophytes (Chen et al., 2023). Therefore, compared to the single variables of nutrient or light conditions, an integration with water depth may provide a better explanation on changes in the collapse and recovery of submerged macrophytes for lakes with large depth gradients.

To explore the thresholds of the collapse and recovery of submerged macrophytes for lakes under large depth gradients, nine lakes with a wide range of water depth and nutrient status in Yunnan Plateau, southwestern China, were investigated. Our purposes were fourfold: 1) To compare the responses of submerged macrophyte biomass and species richness to single variables of nutrients (or turbidity) or transparency and their integration with water depth; 2) To compare the single variable of nutrients (or turbidity) or transparency, a multiplication of nutrients (or turbidity) with water depth and a division of transparency by water depth on the explanation of changes in submerged macrophytes species richness and biomass for lakes with large depth gradients; 3) To explore the thresholds of collapse and recovery of submerged macrophytes for lakes with large depth gradients. The findings of this study can provide threshold conditions for lake managers to restore submerged macrophytes for lakes with large depth gradients.

## 2 Materials and methods

### 2.1 Study area

**Figure 1:**
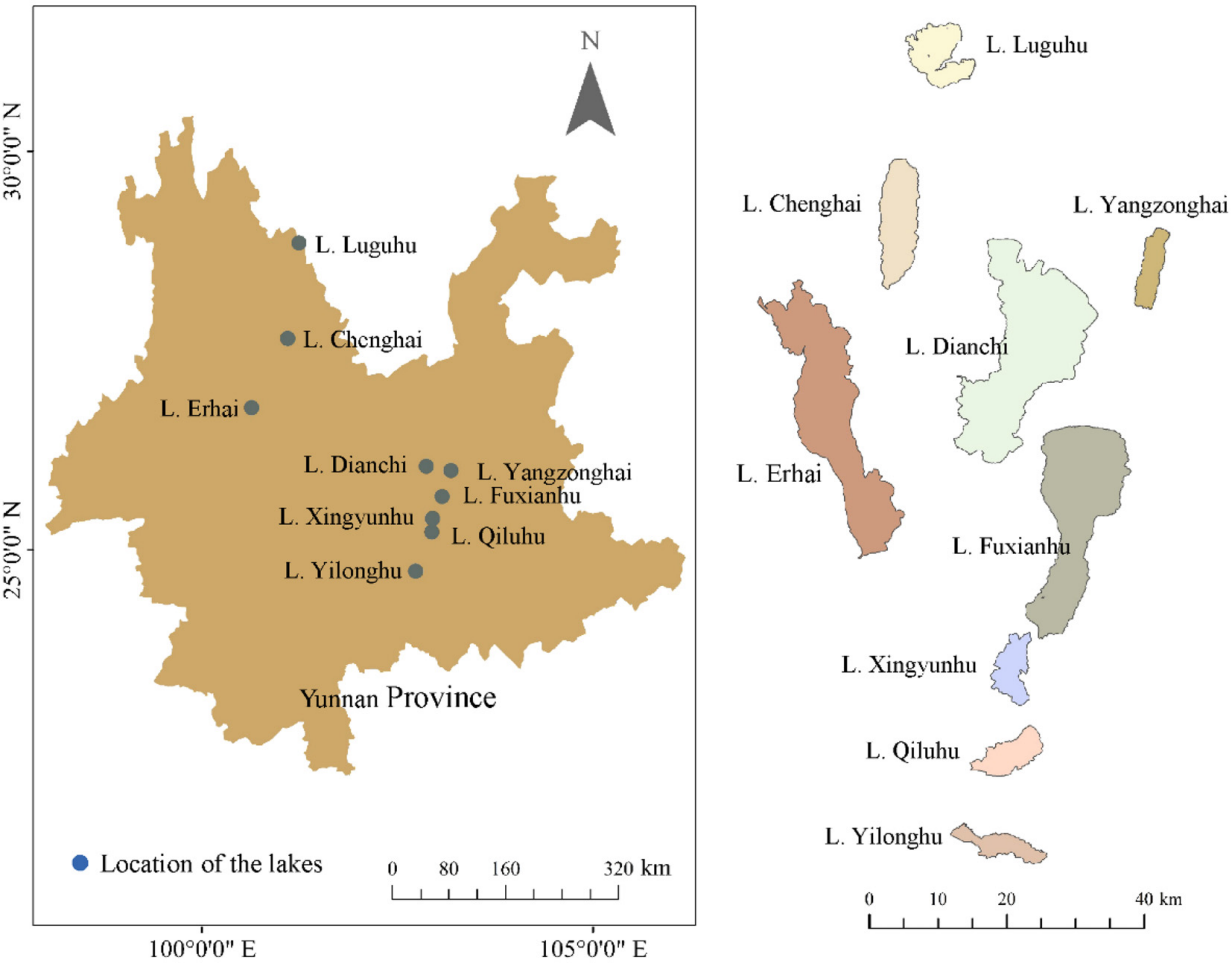
Locations of the studied lakes.

The Yunnan Plateau is located in the southwest of China, dominated by a subtropical highland monsoon climate with an average annual temperature of 15-18 °C and an annual precipitation of 1,000-1,200 mm. Nine plateau lakes (Lake Luguhu, Lake Chenghai, Lake Yangzonghai, Lake Erhai, Lake Dianchi, Lake Fuxianhu, Lake Xingyunhu, Lake Qiluhu, and Lake Yilonghu) (**Figure 1**) were investigated from October to November (a season with high biomass), 2021. The total water area of these nine plateau lakes is approximately 1,021 km^2^, and the total drainage area is 8,110 km^2^. The lake area and mean water depth rangefrom 31.7 to 297.9 km^2^ and 2.2 to 38.6 m, respectively. The area of Lake Dianchi, Lake Fuxianhu, and Lake Erhai are larger than 200 km^2^, and the remaining 6 lakes are smaller than 80 km^2^. Besides Lake Luguhu and Lake Chenghai, all the other lakes have inflowing and outflowing rivers (Yang et al., 2023; Fan et al., 2023).

### 2.2 Field sampling and analysis

The field survey included 226 sampling sites, ranging from 17 to 50 for each lake according to their surface area. Water temperature (WT), dissolved oxygen (DO), conductivity (Cond), and pH were measured *in situ* using a YSI Pro Plus (Yellow Spring Inc, USA). Turbidity (Turb) was measured using a turbidimeter (2100 Q, HACH, Loveland, CO, USA). Water depth (Z) was measured using a depth sounder (Speedtech, SM-5A, USA). Transparency was measured with a Secchi Disc. Depth-integrated water samples were taken from three layers (surface, middle, and bottom water depth) within each site using a 5 L polymethyl methacrylate water sampler and then pooled into a bucket. One liter of well-mixed water was taken back to the laboratory and stored at 0°C for analysis of TN, TP, and phytoplankton Chlorophyll *a* (Chl *a*). TN was determined by using an alkaline potassium persulfate digestion-UV spectrophotometric method, and TP was determined through an ammonium molybdate-UV spectrophotometric method (PERSEE, TU-1810, Beijing, China) (Huang et al., 1999). Chl *a* was extracted in 90% acetone at 4°C for 24 h after filtering the water sample through GF/C filters (Whatman, GE Healthcare UK Limited, Buckinghamshire, UK) (Huang et al., 1999). The absorbance values of the acetone extract were then measured at 665 nm and 750 nm by employing a spectrophotometer, both before and after acidification with 10% HCl. Main limnological characteristics (mean ± SD) of the sampling sites with and without macrophytes were showed in **Supplementary Table A1**.

Submerged macrophytes were randomly collected (2-4 replicates) with a grab-type sampler (0.2 m^2^ in sampling area). Samples were rinsed to remove extraneous material such as sticks, macroinvertebrates, and substrates, and the macrophytes were identified to species and counted, to obtain the species richness (SR). The water on surface was wiped off, and the wet weight was measured. All the samples were immediately brought back to the lab for further analysis.

### 2.3 Data processing and analysis

The biomass of submerged macrophytes (B_Mac_) was expressed by the dry biomass of leaves and stems for vascular plants (known as above-ground biomass). To prevent invalid values when taking logarithms, 0.1 was added to the biomass and species richness of submerged macrophytes (Bachmann et al., 2002). The ‘diffslope’ function of the ‘Simba’ package in R was used to calculate the differences in slope and intercept between the two linear regressions of sites with and without macrophytes (Nekola and White, 1999). Mantel tests were used to explore the potential link between the submerged macrophyte biomass and species richness and nutrients or light conditions and their multiplication with water depth. Spearman’s correlation analysis identified the relevance between nutrients, light conditions, and their multiplications with water depth. Analysis of these processes was completed through the ‘linkET’ package in R. Redundancy discriminant analysis (RDA) and Monte Carlo permutation tests (permutations = 999) were used to analyze the effects of environmental factors on submerged macrophyte biomass and species richness using the ‘vegan’ package (Oksanen et al., 2016). All data analyses were conducted in R 4.1.3 (R Core Team, 2021).

## 3 Results

### 3.1 Environmental variables and submerged macrophytes

**Figure 2:**
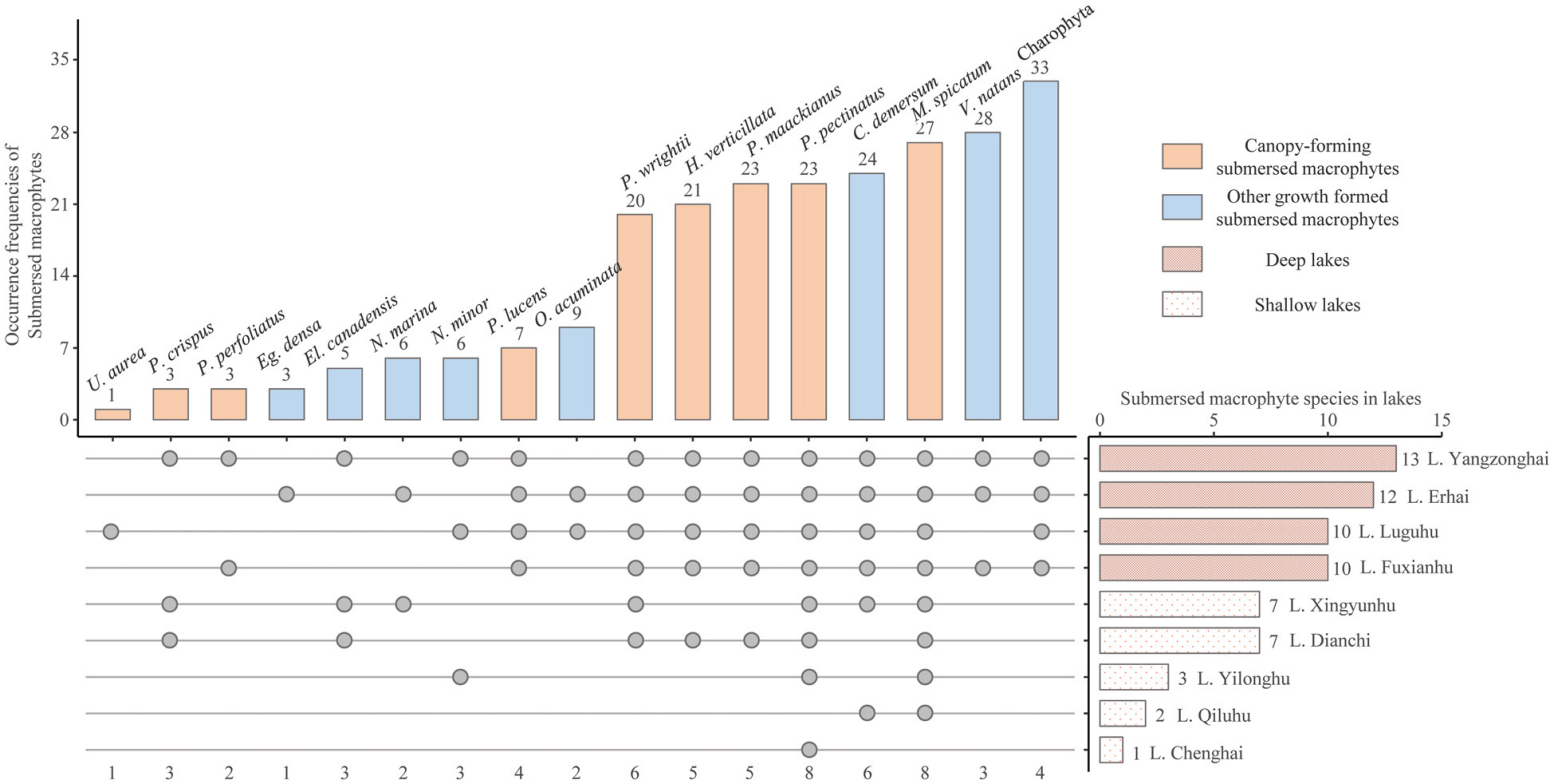
The occurrence frequencies of identified submerged macrophytes in the nine studies lakes (The points indicate the presence of submerged macrophyte species in the lakes. *U. aurea*, *Utricularia aurea*; *P. crispus*, *Potamogeton crispus*; *P. perfoliatus*, *Potamogeton perfoliatus*; *Eg. Densa*, *Egeria densa*; *El. Canadensis*, *Elodea canadensis*; *N. marina*, *Najas marina*; *N. minor*, *Najas minor*; *P. lucens*, *Potamogeton lucens*; *O. acuminata*, *Ottelia acuminata*; *P. wrightii*, *Potamogeton wrightii*; *H. verticillata*; *Hydrilla verticillata*; *P. maackianus*, *Potamogeton maackianus*; *P. pectinatus*, *Potamogeton pectinatus*; *C. demersum*, *Ceratophyllum demersum*; *M.* sp*icatum*, *Myriophyllum* sp*icatum*; *V. natans*, *Vallisneria natans*).

The nutrient concentrations of the studied lakes formed a clear gradient (**Table 1**). The water depth of the sampling sites ranged from 1.8 m to 24.2 m. Submerged macrophytes occurred in 74 of the 226 sampling sites. In total 17 species of submerged macrophytes were identified (**Figure 2**), with 8 species having a frequency higher than 20 and 4 lakes with ≥ 10 species, Charophyta the highest (33) while *Utricularia aurea* the lowest (1) according to their occurrence frequency of sampling sites. The occurrence frequencies of canopy-forming submerged macrophytes were higher than others. Species richness of submerged macrophytes in deep lakes was higher than that in shallow lakes. According to the numbers of distributed lakes, *Potamogeton pectinatus* and *Myriophyllum spicatum* were most widely distributed in 8 lakes (**Figure 2**).

**Table 1:** Main limnological characteristics (mean ± SD) of the sampling sites in nine studied lakes.

Studied lakes	Area (km^2^)	Z_Max_ (m)	Z_Min_ (m)	Z_M_ (m)	TN (mg/L)	TP (mg/L)	Chl *a* (µg/L)	Turb (NTU)
L. Fuxianhu	216.6	14.3	1.9	9.1 ± 4.5	0.13 ± 0.04	0.004 ± 0.002	1.93 ± 0.46	0.6 ± 0.2
L. Luguhu	48.5	69.4	1.3	24.2 ± 22.0	0.11 ± 0.02	0.006 ± 0.002	0.76 ± 0.18	0.7 ± 0.2
L. Chenghai	77.2	26.8	4.7	19.7 ± 7.2	0.56 ± 0.12	0.03 ± 0.01	8.54 ± 5.55	2.0 ± 0.8
L. Yangzonghai	31.7	27.5	0.5	14.4 ± 9.8	0.77 ± 0.06	0.03 ± 0.006	6.58 ± 3.96	2.1 ± 0.7
L. Erhai	249.0	20.0	0.8	7.2 ± 4.9	0.99 ± 0.85	0.05 ± 0.06	32.54 ± 31.54	3.5 ± 3.1
L. Dianchi	297.9	6.7	0.4	4.5 ± 1.7	2.00 ± 0.43	0.06 ± 0.04	44.07 ± 25.81	27.4 ± 7.2
L. Xingyunhu	34.7	10.3	1.3	5.9 ± 3.0	1.81 ± 0.40	0.08 ± 0.03	51.91 ± 46.86	18.6 ± 14.8
L. Yilonghu	38.0	4.5	0.7	1.8 ± 0.9	3.22 ± 0.21	0.10 ± 0.01	84.00 ± 10.72	48.6 ± 11.1
L. Qiluhu	36.9	6.0	1.4	3.5 ± 4.6	4.00 ± 0.87	0.14 ± 0.01	83.06 ± 14.60	21.5 ± 13.0

Area, the area of the studied lakes; Z_M_, the mean water depth; Z_Max_, the maximum water depth; Z_Min_, the minimum water depth; TN, total nitrogen; TP, total phosphorus; Chl *a*, phytoplankton chlorophyll *a*; Turb, turbidity.

### 3.2 The environment ranges of eight frequent submerged macrophytes

**Figure 3:**
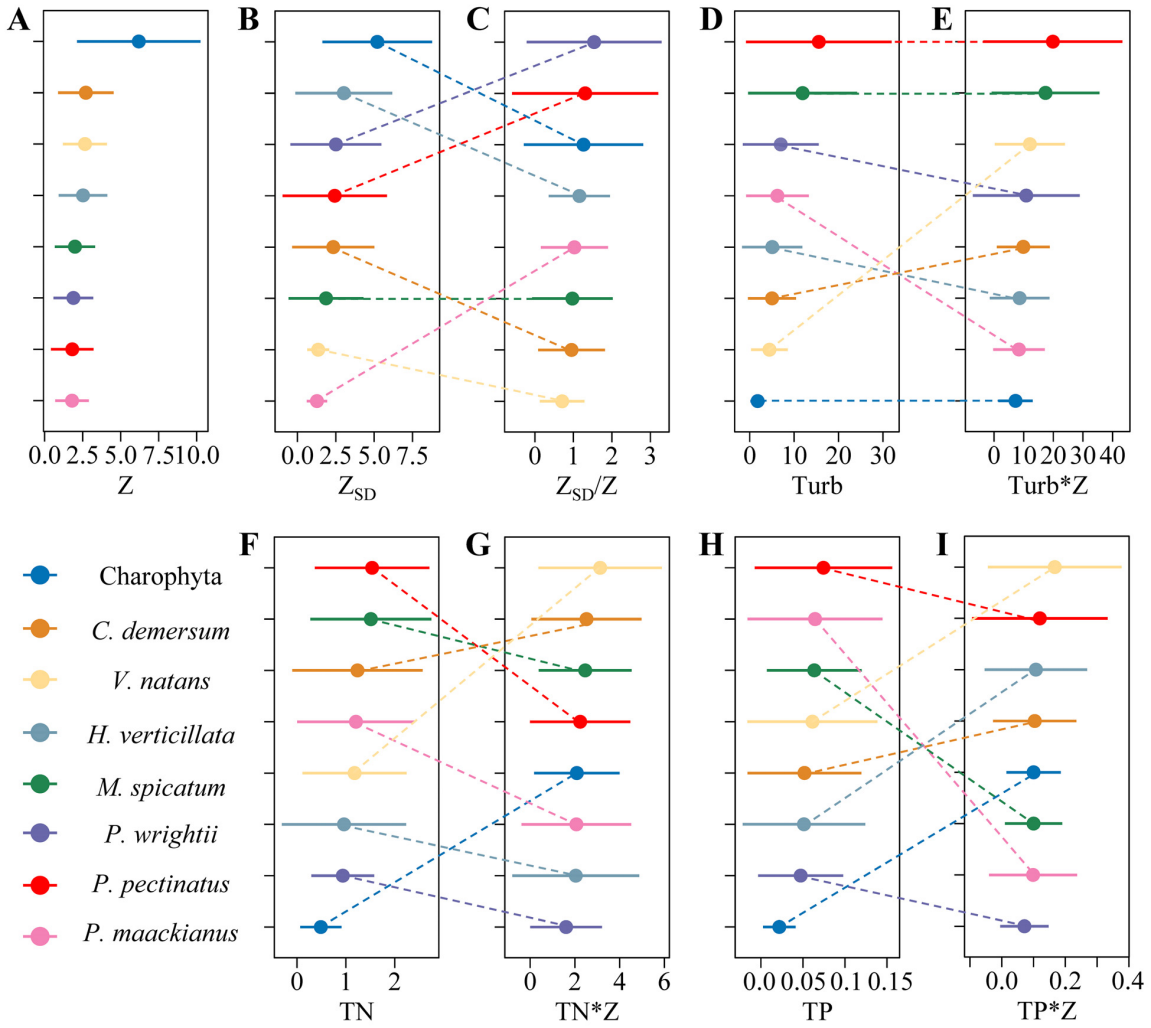
The distribution ranges of the eight frequent submerged macrophytes along the gradients of **(A)** water depth, **(B)** transparency, **(D)** turbidity, **(F)** TN - total nitrogen, **(H)** TP - total phosphorus and along most of them integrated with water depth e.g., **(C)** transparency, **(E)** turbidity, **(G)** TN, **(I)** TP (*C. demersum*, *Ceratophyllum demersum*; *V. natans*, *Vallisneria natans*; *H. verticillata*; *Hydrilla verticillata*; *M.* sp*icatum*, *Myriophyllum* sp*icatum*; *P. wrightii*, *Potamogeton wrightii*; *P. pectinatus*, *Potamogeton pectinatus*; *P. maackianus*, *Potamogeton maackianus*).

The distribution of submerged macrophytes is wide across environmental gradients. The maximum growing depth of Charophyte was the deepest (12.5 m) and the distribution was the widest (0.48-12.5 m), there were no significant differences for other species (**Figure 3A**). Besides, the maximum transparency of the distribution of Charophyte was the largest and the distribution range was also the widest. However, when integrated with water depth, the maximum Z_SD_/Z of the distribution of *Potamogeton wrightii* was the largest among the eight frequent submerged macrophytes and the distribution range of *P*. *pectinatus* along the gradients of Z_SD_/Z was the widest range (**Figures 3B, C**). In general, the distributions of the canopy-forming submerged macrophytes, i.e. *Potamogeton pectinatus* and *Myriophyllum spicatum* had larger and wider range of Turb, TN, and TP, which were the lowest for Charophyte. When integrated with water depth, the distribution range of the eight frequently submerged macrophytes along the gradients of environmental factors changed greatly. For example, When Z_SD_ was combined with water depth, the distribution range of *Potamogeton maackianus* along the Z_SD_/Z gradients expanded notably. Conversely, when Turb, TN, and TP were integrated with water depth, this range contracted significantly. *Vallisneria natans* had no greater changes when Z_SD_ integrated with water depth, but increased a lot when Turb, TN, and TP integrated with water depth (**Figure 3**).

### 3.3 Relationships of Turb, Z_SD_, and Chl a with nutrients for sampling sites with and without macrophytes

**Figure 4:**
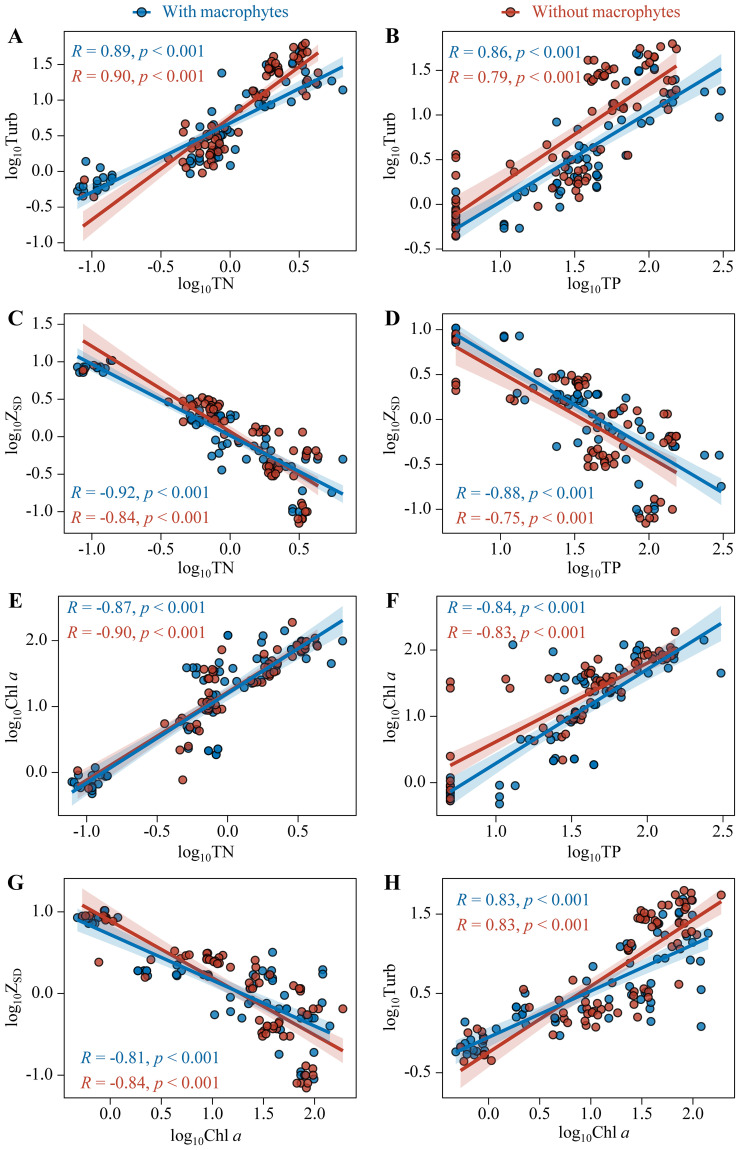
Relationships of Turb **(A, B)**, Z_SD_ **(C, D)**, and Chl *a* **(E, F)** with TN and TP and relationships of Z_SD_ **(G)** and Turb **(H)** with Chl *a* for sampling sites with (Blue points) and without macrophytes (Red points).

The ecosystem states, i.e. Turb, Z_SD_, and Chl *a*, all showed significantly positive relationships with TN and TP for sampling sites with and without macrophytes (*p* < 0.001). Besides, Turb and Z_SD_ also showed significantly positive relationships with Chl *a* (*p* < 0.001). But there were no notable differences in the relationships between sampling sites with and without macrophytes, only found significant difference in the relationship of Turb with TN between sampling sites with and without macrophytes. The scatterplots for sampling sites with and without macrophytes overlapped along the full gradients of x-variables; the pairs of sampling sites mixed together for the relations of log_10_Turb, log_10_Z_SD_, and log_10_Chl *a* against the nutrients and the relations of log_10_Turb and log_10_Z_SD_ against log_10_Chl *a* (**Figure 4**).

### 3.4 Relationships between submerged macrophyte biomass, species richness and environmental factors

**Figure 5:**
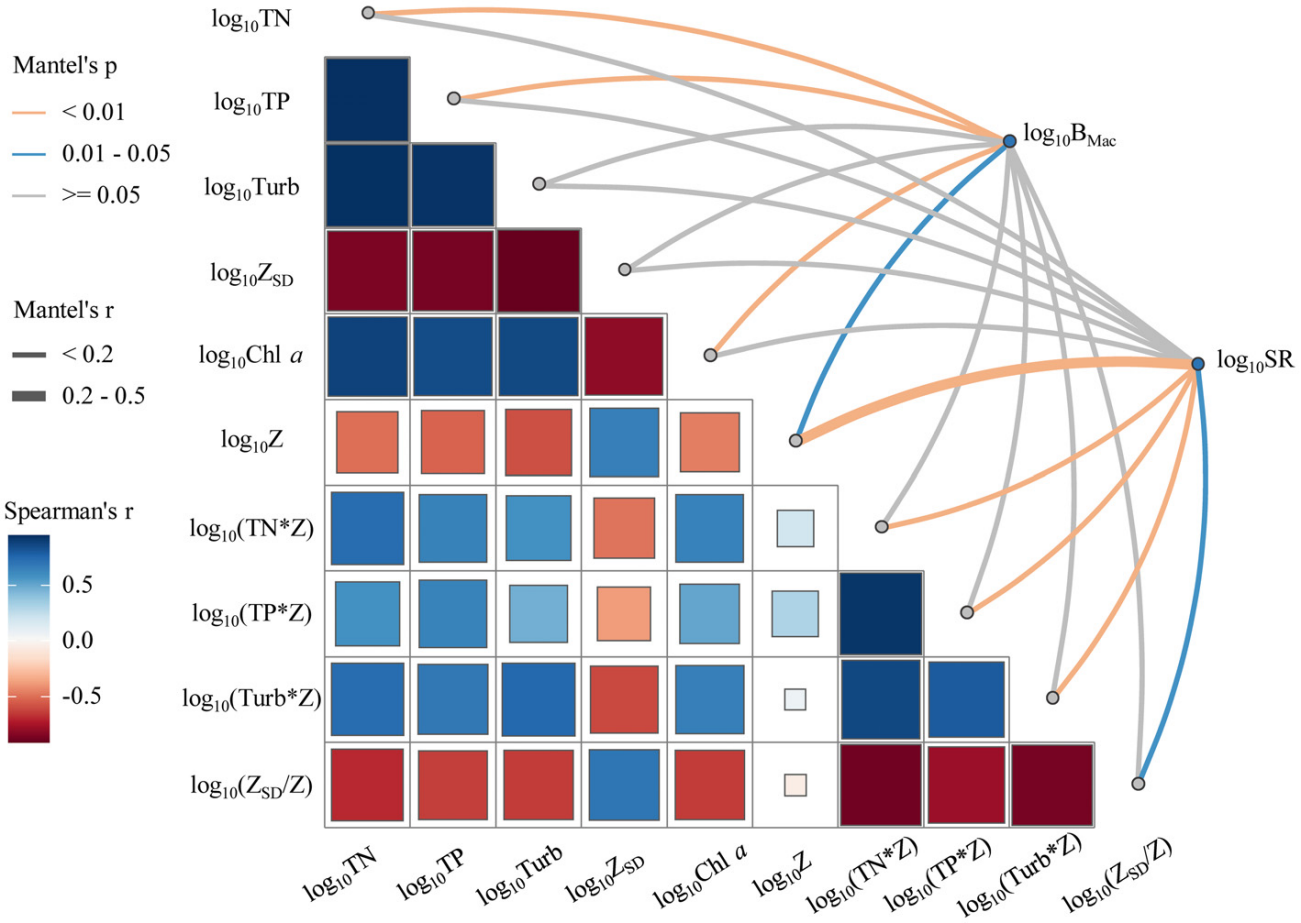
Pairwise correlations of environmental factors are shown with a color gradient denoting Spearman’s correlation coefficient. Submerged macrophytes biomass (B_Mac_) and species richness (SR) were related to each environmental factor by Mantel tests (The size of the square stands for the size of the Spearman’s correlation coefficient).

Mantel analysis showed that the biomass of submerged macrophytes (B_Mac_) had highly positive correlations with Z and negative correlations with TN, TP, and Chl *a* (*p* < 0.05, **Figure 5**), while the species richness of submerged macrophytes (SR) indicated positive correlations with Z_SD_/Z and negative correlations with Z, TN*Z, TP*Z, and Turb*Z (*p* < 0.05, **Figure 5**).

**Figure 6:**
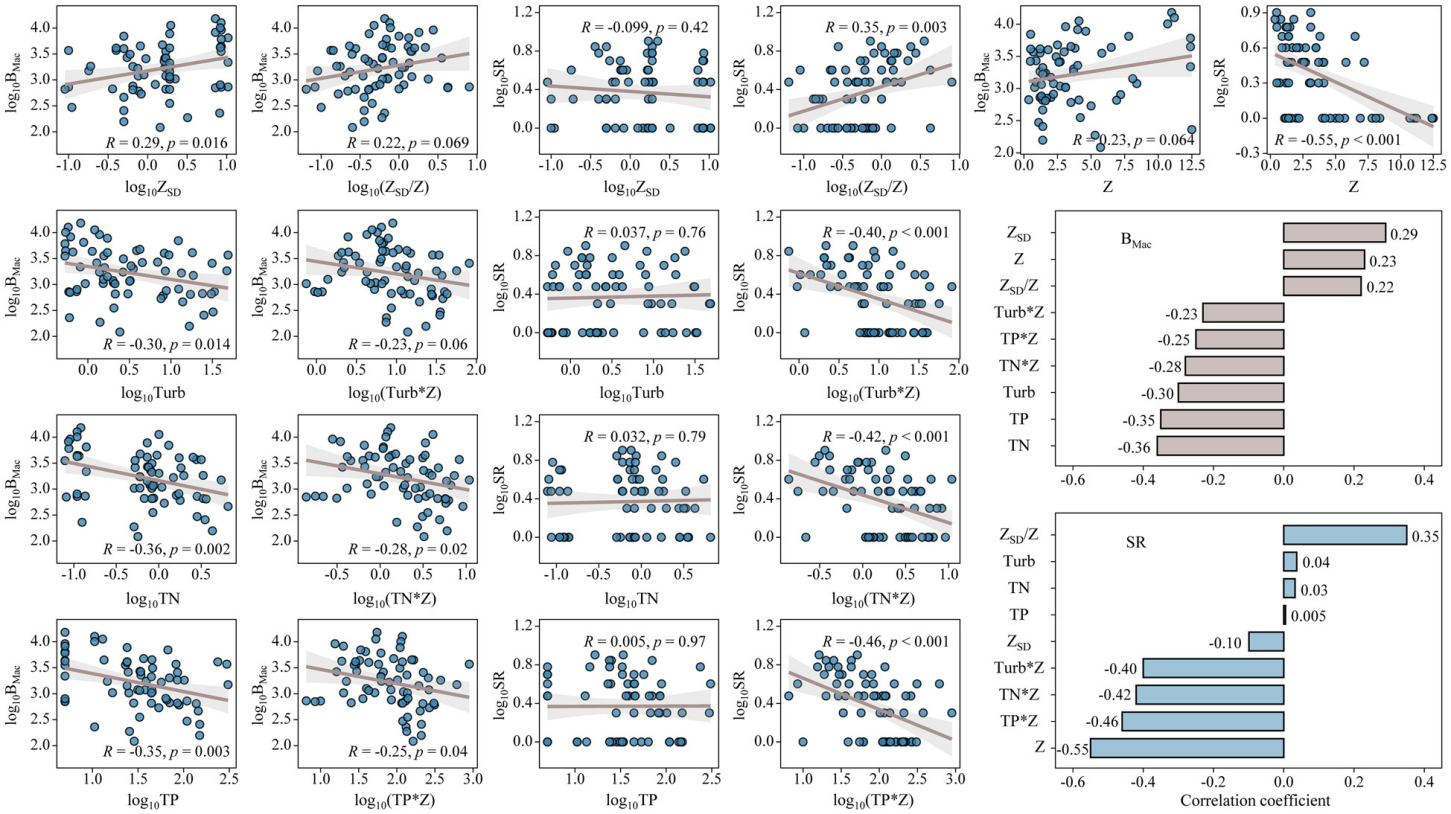
Linear regressions between submerged macrophyte biomass (B_Mac_) and species richness (SR) and Z_SD_, Turb, TN, TP, Z_SD_/Z, Turb*Z, TN*Z, and TP*Z. Histograms showed the values of correlation coefficient (*R*) of biomass (B_Mac_) and species richness (SR) with Z_SD_, Turb, TN, TP, Z_SD_/Z, Turb*Z, TN*Z, and TP*Z.

As for regression analysis, the biomass of submerged macrophytes increased remarkably with increasing Z_SD_ (*p* < 0.05, **Figure 6**), while decreased significantly with growing Turb, TN, and TP (*p* < 0.05, **Figure 6**). However, it showed no relationships with Z. The biomass of submerged macrophytes also indicated same trends with multiplications of nutrient concentrations (or turbidity) with water depth and a division of transparency by water depth, although only obvious relationships were found with TN*Z and TP*Z (*p* < 0.05, **Figure 6**). The species richness of submerged macrophytes showed no relationships with Z_SD_, Turb, TN, and TP, while increased significantly with growing Z_SD_/Z (*p* < 0.01, **Figure 6**) and saw a notable decline with increasing Z, Turb*Z, TN*Z, and TP*Z (*p* < 0.01, **Figure 6**).

The histograms showed the correlation coefficient (*R*) of biomass and species richness with Z, Z_SD_, Turb, TN, TP, Z_SD_/Z, Turb*Z, TN*Z, and TP*Z. The absolute values of *R* between B_Mac_ and Z_SD_, Turb, TN, and TP were higher than that when Z_SD_, Turb, TN, and TP integrated with water depth, however, the absolute values of *R* between SR and Z_SD_, Turb, TN, and TP were less than that when Z_SD_, Turb, TN, and TP integrated with water depth (**Figure 6**).

**Figure 7:**
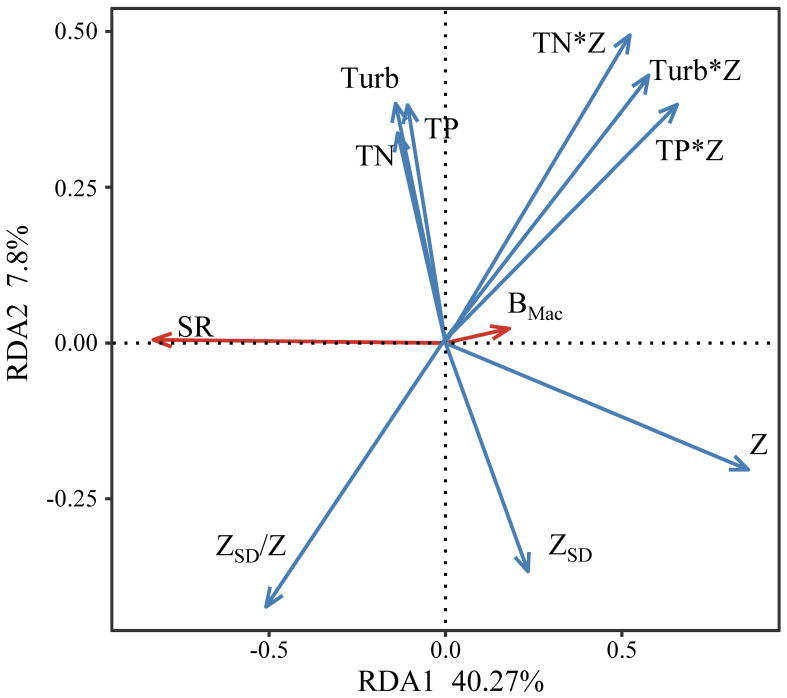
Redundancy analysis (RDA) of environmental factors and submerged macrophyte biomass (B_Mac_) and species richness (SR).

The redundancy analysis revealed that 48.07% of the overall variability was explained by the first two principal components (RDA1 and RDA2) (**Figure 7**). Among the assessed physicochemical factors, Z was the most significant variable that influenced submerged macrophyte biomass and species richness (*p* < 0.001).

### 3.5 The thresholds of collapse and recovery of submerged macrophytes

**Figure 8:**
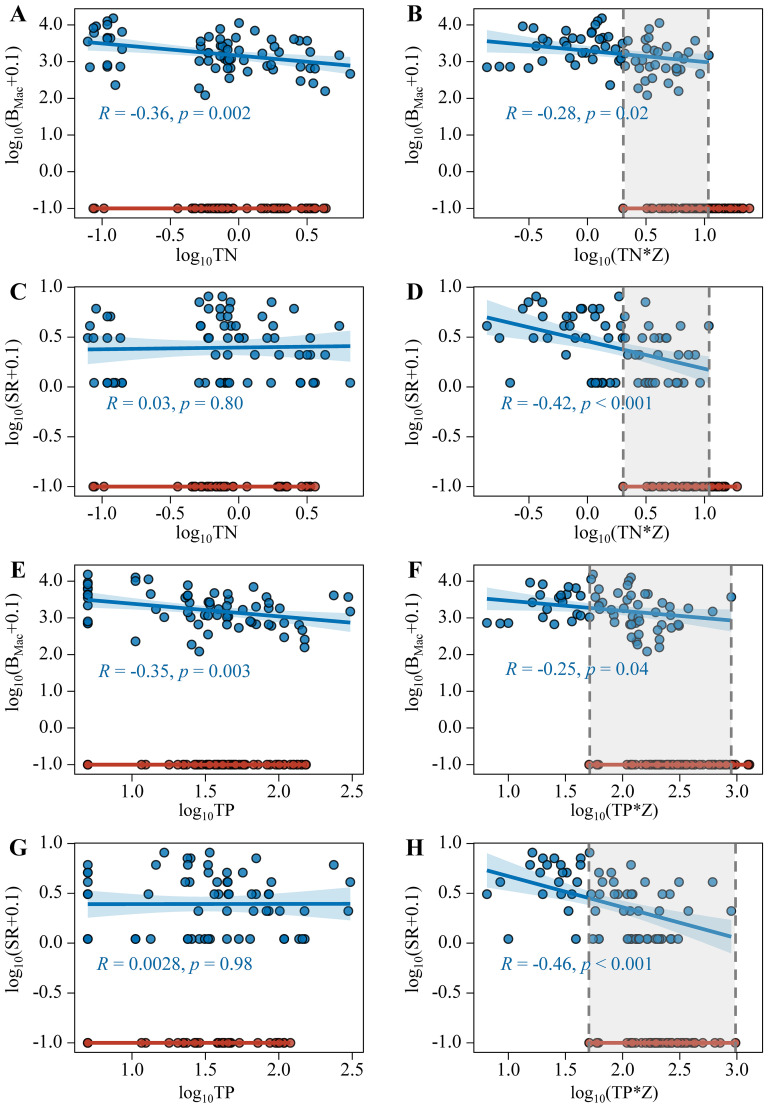
Relationships between log_10_TN **(A, C)**, log_10_(TN*Z) **(B, D)**, log_10_TP **(E, G)**, log_10_(TP*Z) **(F, H)** with log_10_(B_Mac_+0.1) and log_10_(SR+0.1) (Blue points indicate the sampling sites with macrophytes, red points indicate the sampling sites without macrophytes).

When plotting the submerged macrophyte biomass and species richness against Z_SD_ and Turb, no folded bifurcation was found. The scatterplots for sampling sites with and without macrophytes overlapped along the full gradients of x-variables (**Figures 8A, C, E, G**). While multiplying Z_SD_ and Turb by water depth, a clear folded bifurcation emerged for their relations with log_10_(B_Mac_+0.1) and log_10_(SR+0.1) (**Figures 8B, D, F, H**).

The thresholds of collapse and recovery of submerged macrophytes in lakes with large water depth spans were 0.06 and 0.53 for Z_SD_/Z, 81.6 and 9.92 NTU m for Turb*Z, respectively.

## 4 Discussion

The study found that *Potamogeton pectinatus* and *Myriophyllum spicatum* were most widely distributed in the investigated lakes, with a larger nutrient and turbidity ranges. In other words, they had a higher resistance to high nutrients and turbidity, which was consistent with other survey (Li et al., 2017). *Potamogeton pectinatus* and *Myriophyllum spicatum* both belongs to canopy-forming submerged macrophytes with long branches and vigorous crown. Due to the high growth rate, canopy-forming submerged macrophytes can concentrate at or near the water surface and have strong competition ability for light and space (Chambers and Kalff, 1987; James et al., 2004). Compared with other growth forms, canopy-forming macrophytes have a high tolerance to eutrophication (Zhang et al., 2022b). As a result, when the water quality deteriorates, canopy-forming submerged macrophytes gradually replace other growth forms as the main dominant species (Jeppesen et al., 2000; Sand-Jensen et al., 2017; Murphy et al., 2018).

Our study further found that submerged macrophyte species richness was more susceptible to the combinations of nutrient (or turbidity) or light conditions with water depth than single variables of nutrient or light conditions, while biomass showed the opposite patterns. The main reason is that water depth had a substantial negative effect on species richness, with no impact on biomass, which was in accordance with other previous study (Fu et al., 2014). Water depth can more directly bring about light reduction and the stress of water pressure of submerged macrophytes (Schwarz et al., 1996; Søndergaard et al., 2013). With the increase of water depth and the attenuation of light, canopy-forming species, i.e. *P*. *pectinatus* and *P*. *maackianus* with higher light demand cannot survive and are replaced by bottom-dwelling and rosette-forming species with low light requirement. In our study, Charophyta was the only taxon where the water was deeper than 7 m. Due to the absence of competition from other species for space and light in deep water, charophytes were able to proliferate in large numbers and formed dense and extensive “underwater meadows” with enormous biomass (Middelboe and Markager, 1997). Therefore, although the numbers of species in submerged macrophyte community decreased as the water depth increased, the overall biomass showed no obvious reduction.

Our study found that the thresholds of collapse and recovery of submerged macrophytes in deep lakes were 0.06 and 0.53 for Z_SD_/Z, 81.6 and 9.92 NTU m for Turb*Z, respectively. Compared to Yangtze River Plain lakes, the thresholds for Z_SD_/Z of collapse of submerged macrophytes in Yunnan Plateau lakes were much lower than that in Yangtze River Plain lakes (Yunnan Plateau lakes: 0.06 and Yangtze River Plain lakes: 0.45). It seemed that the submerged macrophytes in Yunnan Plateau lakes had a higher resistance to low light than those in Yangtze River Plain lakes. The higher stoichiometric characteristics of submerged macrophytes in Yunnan Plateau lakes can best explain it (Xing et al., 2013; Li et al., 2017). For instance, the contents of C, N, and P of *M*. *spicatum* in Yangtze River Plain lakes were 359.87, 12.09, and 2.92 mg g^-1^, respectively, while the contents were 425.11, 22.84, and 3.06 mg g^-1^ in Yunnan Plateau lakes. The similar values were also found for *P*. *malaianus* (Xing et al., 2013; Li et al., 2017). Under stresses, plants can produce various kinds of primary and secondary metabolites, i.e. enzyme to protect themselves, while, C, N, and P are the basic elements for the synthesis of various enzymes. Submerged macrophytes may accumulate additional C, N, and P to improve their resistance to low light. Besides, the difference in the UV part of the spectrum between the Yunnan Plateau and Yangtze plain may also influence the distribution of submerged macrophytes. UV-B radiation, which is harmful to organisms, can penetrate several meters or even tens of meters into the water column (Smith et al., 1992). Due to the negative effects of strong UV-B on the physiological characteristics of submerged macrophytes, macrophytes may shift to greater depths to avoid damage (Yuan et al., 2019). Therefore, the collapse thresholds for Z_SD_/Z of submerged macrophytes in Yunnan Plateau lakes were much lower than those in Yangtze River Plain lakes.

Previous studies on the thresholds of disappearance of submerged macrophytes was commonly focused on shallow lakes (Bayley and Prather, 2003; Ibelings et al., 2007; Wang et al., 2014). It had been identified turbidity and transparency as key environmental factors, which influenced the survival of submerged macrophytes in shallow lakes (Scheffer et al., 1993; Wang et al., 2014; van Wijk et al., 2023). However, due to nonnegligible effect of water depth on the survival of submerged macrophytes in deep lakes (Jeppesen et al., 2007; Kosten et al., 2009), the thresholds in deep lakes were different from shallow lakes (Jeppesen et al., 1990; Bachmann et al., 2002; Kosten et al., 2009; Wang et al., 2014), which may mainly depend on the differences of lake morphometry (Genkai-Kato and Carpenter, 2005; Kosten et al., 2009). For instance, the average water depth in this study was 10.03 m (1.79-24.19 m), which is much deeper than other lakes such as Lake Væng (1.2 m), Lake Veluwe (1.5m), and Lake Baoan (1.9 m) (Jeppesen et al., 1999; Ibelings et al., 2007; Wang et al., 2014). Because of the good variation of B_Mac_ along the gradients of Z_SD_/Z and Turb*Z (contrary to the situation when water depth was removed), the thresholds of collapse and recovery of submerged macrophytes in deep lakes had a great dependence on the water depth. However, there was also a certain relevance between the thresholds for the two types of lakes. Turb and nutrients (promoted the growth of phytoplankton and then had a shading effect) can affect underwater light conditions and thus affect the distribution of submerged macrophytes (Schelske et al., 2010; Arthaud et al., 2012; Olsen et al., 2015; Zhang et al., 2016), but water depth can more directly bring about light reduction and the stress of water pressure (Schwarz et al., 1996; Søndergaard et al., 2013). It seemed that underwater light conditions were the most important factors affecting the survival of submerged macrophytes. Turb, Z_SD_, Chl *a*, nutrient, and water depth all restricted the maximum growing depth of submersed macrophytes (Chambers and Kalff, 1985; Søndergaard et al., 2013). Therefore, the survival of submerged macrophytes in lakes with different depths mainly depended on the demand of light.

## 5 Conclusions

In the present study, we found that: 1) Canopy-forming submerged macrophytes, i.e. *Potamogeton pectinatus* and *Myriophyllum spicatum*, were most widely distributed in the investigated lakes, with a higher resistance to high nutrients and turbidity; 2) Submerged macrophyte species richness had significant negative response to water depth, while biomass did not; 3) Compared to the single variables, a multiplication of turbidity and a division of transparency with water depth provided a better explanation on predicting the thresholds of collapse and recovery of submerged macrophytes for lakes with large depth gradients.; 4) The thresholds of Z_SD_/Z were 0.06 for the collapse of submerged macrophytes and 0.53 for the recovery of submerged macrophytes; the corresponding thresholds were 81.6 and 9.92 NTU m for Turb*Z, respectively. Our findings demonstrate that the role of water depth should be taken into account when restoring submerged macrophytes in the management of lakes with large depth gradients.

## Data Availability

The original contributions presented in the study are included in the article/**Supplementary Material**. Further inquiries can be directed to the corresponding authors.
