# Supplementary material for: Integrating water depth to predict the threshold of collapse and recovery of submerged macrophytes for lakes with large depth gradients
Source: Front Plant Sci. 2025 Feb 24;16:1541394. doi: 10.3389/fpls.2025.1541394 (PMC11891350; doi:10.3389/fpls.2025.1541394)
Supplement: Supplementary file 1 [file DataSheet1.docx]

**Integrating water depth to predict the threshold of collapse and recovery of submerged macrophytes for lakes with large depth gradients**

Yexin Yu ^a, b 1^, Yehao Li ^a, 1^, Haijun Wang ^a, c,^ *, Haojie Su ^a,^ *, Qingyang Rao ^a^, Ying Liu ^a^, Ping Xie ^a, d^

*^a^ Institute for Ecological Research and Pollution Control of Plateau Lakes, School of Ecology and Environmental Science; Yunnan Key Laboratory of Ecological Protection and Resource Utilization of River-lake Networks; Qilu Lake Field Scientific Observation and Research Station for Plateau Shallow Lake in Yunnan Province; Ministry of Education Key Laboratory for Transboundary Ecosecurity of Southwest China, Yunnan University, Kunming 650500, China*

*^b^ Institute of International Rivers and Eco-Security, Yunnan University, Kunming 650500, China*

*^c^ Institute of Yunnan Plateau Indigenous Fish, Kunming 652115, China*

*^d^ Donghu Experimental Station of Lake Ecosystems, State Key Laboratory of Freshwater Ecology and Biotechnology, Institute of Hydrobiology, Chinese Academy of Sciences, Wuhan, 430072, China*

* Corresponding author

Email: wanghaijun@ynu.edu.cn (Haijun Wang), suhaojie@ ynu.edu.cn (Haojie Su)

^1^ These authors share the first authorship

Supplementary Material

**Table A1** Main limnological characteristics (mean ± SD) of the sampling sites with and without macrophytes.

|  | Sampling sites  with macrophytes | Sampling sites  without macrophytes |
| --- | --- | --- |
| TN | 1.22±1.32^a^ | 1.65±1.16^b^ |
| TP | 0.05±0.06^a^ | 0.05±0.04^a^ |
| Chl *a* | 30.94±37.60^a^ | 35.80±35.02^a^ |
| Turb | 7.80±11.08^a^ | 15.46±18.53^b^ |
| Z | 3.79±3.34^a^ | 13.12±13.03^b^ |
| Z_SD_ | 3.01±3.30^a^ | 1.85±2.25^b^ |
| WT | 18.62±2.02^a^ | 17.30±2.70^a^ |
| DO | 8.24±6.21^a^ | 7.50±1.83^a^ |
| Cond | 202.90±119.77^a^ | 197.64±295.11^a^ |
| pH | 8.49±0.30^a^ | 8.48±0.29^a^ |
| ORP | 179.61±78.87^a^ | 143.51±79.68^a^ |

Note: TN, total nitrogen; TP, total phosphorus; Chl *a*, phytoplankton chlorophyll *a*; Turb, turbidity; Z, water depth; Z_SD_, transparency; WT, water temperature; DO, dissolved oxygen; Cond, conductivity; ORP, oxidation-reduction potential.

**
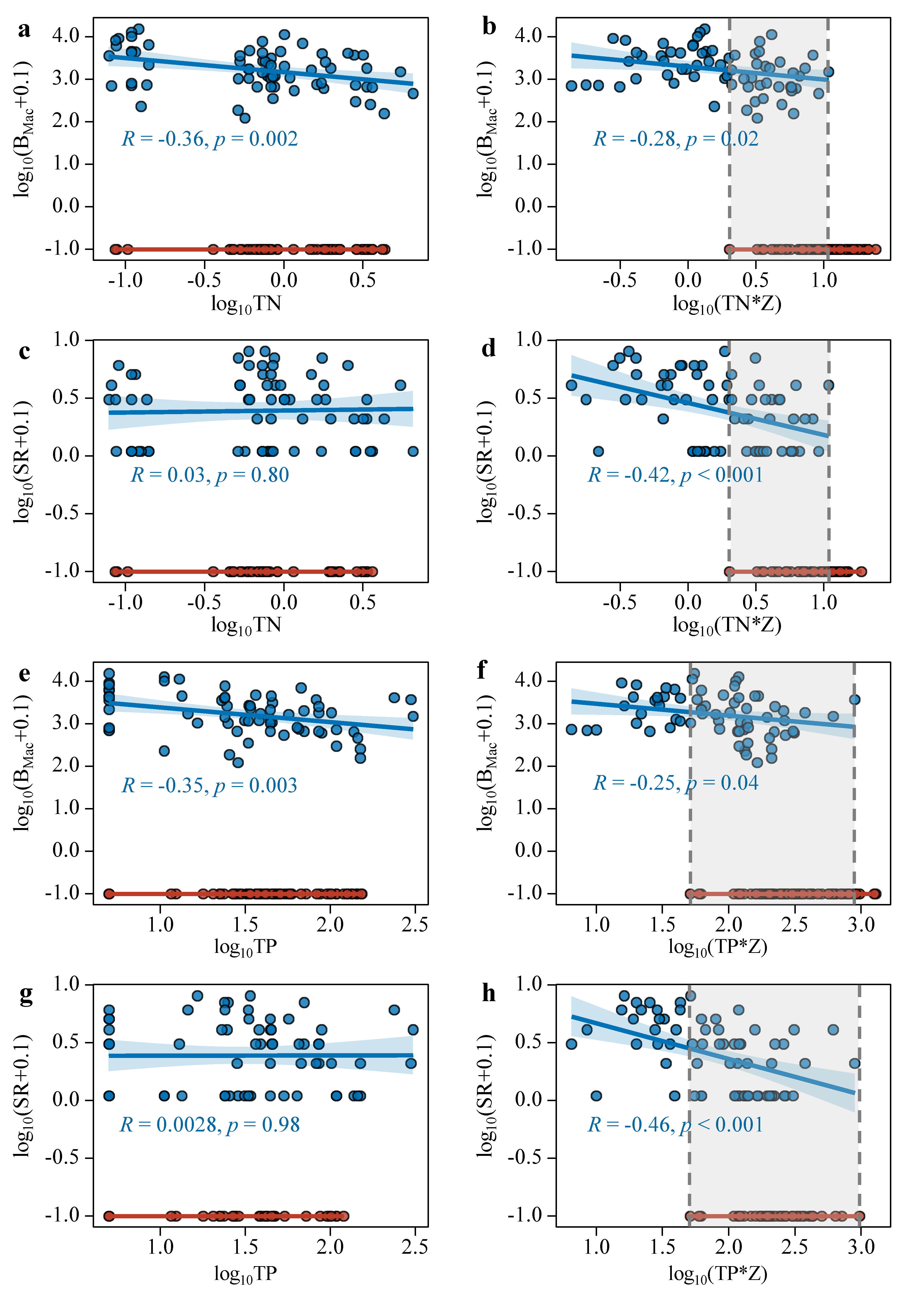
**

Figure S1 Relationships between log_10_TN (a, c), log_10_(TN*Z) (b, d), log_10_TP (e, g), log_10_(TP*Z) (f, h) with log_10_(B_Mac_+0.1) and log_10_(SR+0.1) (Blue points indicate the sampling sites with macrophytes, red points indicate the sampling sites without macrophytes).
